# Postoperative recurrence in locally advanced rectal cancer: how does neoadjuvant treatment affect recurrence pattern?

**DOI:** 10.1186/s12957-023-03136-0

**Published:** 2023-08-16

**Authors:** Ryosuke Okamura, Yoshiro Itatani, Yusuke Fujita, Nobuaki Hoshino, Shintaro Okumura, Kazuhiro Nishiyama, Koya Hida, Kazutaka Obama

**Affiliations:** Department of Surgery, Kyoto University Hospital, 54 Shogoin-Kawahara-Cho, Sakyo-ku, Kyoto, 606-8507 Japan

**Keywords:** Rectal cancer, Recurrence, Neoadjuvant, Metastasectomy

## Abstract

**Background:**

The treatment strategy for locally advanced rectal cancer (LARC) has recently expanded from total mesorectal excision to additional neoadjuvant chemoradiotherapy (nCRT) and/or systemic chemotherapy (NAC). Data on disease recurrence after each treatment strategy are limited.

**Methods:**

Clinical stage II to III rectal cancer patients who underwent curative surgery between July 2005 and February 2021 were analyzed. The cumulative incidence and site of first recurrence were assessed. The median follow-up duration was 4.6 years.

**Results:**

Among the 332 patients, we performed nCRT and NAC in 15.4% (*N*=51) and 14.8% (*N*=49), respectively. The overall recurrence rate was 23.5% (*N*=78). Although several differences in tumor stage or location were observed, there was no significant difference in the rate among the surgery alone (*N*=54, 23.3%), nCRT (*N*=11, 21.6%), and NAC (*N*=13, 26.5%) groups. In this cohort, the local recurrence rate (18.4%) was higher than the rate of distant metastasis in the NAC group (14.3%). All patients with recurrence in the nCRT group had distant metastases (*N*=11: one patient had distant and local recurrences simultaneously). For pathological stage 0-I, the recurrence rate was higher in the nCRT and NAC groups than in the surgery-alone group (nCRT, 10.0%; NAC, 15.4%; and surgery-alone, 2.0%). Curative-intent resection of distant-only recurrences significantly improved patients’ overall survival (hazard ratio [95% confidence interval], 0.34 [0.14–0.84]), which was consistent even when stratified according to neoadjuvant treatment. Regardless of neoadjuvant treatment, >80% of recurrences occurred in the first 2.2 years, and 98.7% within 5 years after surgery.

**Conclusion:**

Regardless of neoadjuvant treatment, detecting distant metastases with intensive surveillance, particularly in the first 2 years after surgery, is important. Also, even if neoadjuvant treatment can downstage LARC to pathological stage 0-I, careful follow-up is needed.

**Supplementary Information:**

The online version contains supplementary material available at 10.1186/s12957-023-03136-0.

## Introduction

Treatment strategies for locally advanced rectal cancer (LARC) have continuously evolved. In Western countries, total mesorectal excision (TME) with neoadjuvant chemoradiotherapy (nCRT) has been the standard treatment for LARC. In recent years, total neoadjuvant therapy (TNT) (i.e., chemoradiotherapy plus consolidation/induction systemic chemotherapy) has also been introduced with less toxicity and better compliance than conventional nCRT followed by TME with postoperative adjuvant chemotherapy [1]. TNT may avoid definitive stoma and postoperative complications [2]. In contrast, in Eastern countries, mainly in Japan, the lateral pelvic lymph node (LPLN) dissection technique without neoadjuvant chemoradiotherapy has traditionally been used to control LPLN metastasis in patients with LARC [3]. Multimodal treatment of LARC is widely used in clinical practice.

Previous literature regarding colon or colorectal cancer showed that over 80% of postoperative disease recurrences occurred in the first 2–3 years after surgery and over 95% occurred in the first 5 years [4–6]. Thus, the clinical guidelines for colorectal cancer recommend routine postoperative surveillance with shorter intervals during the first 2 or 3 years after surgery and 5 years of duration for detecting metachronous disease recurrences at an asymptomatic, preinvasive stage, and treating them immediately although the optimal surveillance strategy remains undefined. Early detection without symptoms leads to early treatment [7, 8]. Even if the recurrent lesion is unresectable or borderline resectable at the time of detection, current advanced chemotherapy may lead to subsequent curative-intent surgery. Therefore, it is important to comprehend the patterns of postoperative recurrence; however, the differences according to neoadjuvant treatment have not been sufficiently addressed. This study focused on patients with LARC and evaluated the incidence patterns and surgical resectability of recurrence.

## Methods

### Study population

We investigated patients with primary LARC at the Kyoto University Hospital (Kyoto, Japan). Patients who underwent curative surgery for clinical stages II to III (AJCC and UICC) (T1-2 N1-2 or T3-4 N any), diagnosed with CT scan and pelvic MRI, between July 2005 and February 2021 were eligible (*N*=337). Only patients with pathologically proven adenocarcinoma were included. According to the Japanese guidelines, LARC is defined as follows: the main lesion of tumor is located in the upper rectum (between the lower level of the S2 vertebra and the peritoneal reflection), lower rectum (between the peritoneal reflection and the upper level of the puborectal sling), and anal canal (between the upper level of the puborectal sling and the anal verge) [9]. Five patients whose metastatic lesions were intraoperatively detected were excluded from the analyses (metastasis to the liver [*N*=3], distant lymph nodes [*N*=1], and peritoneum [*N*=1]). Data regarding clinical and pathological tumor findings, neoadjuvant and adjuvant treatment, first recurrence after surgery for primary LARC, treatment for recurrence, and patients’ survival were extracted from medical records. The study protocol was approved by the Central Institutional Review Board (IRB#, R3768).

### Surgical strategy and postoperative surveillance

The standard surgical procedure for LARC is rectal resection using the TME technique. At our institute, neoadjuvant therapies, nCRT, and systemic chemotherapy (NAC) have been selectively added [10]. Usually, long-course chemoradiotherapy combining radiation (45–50 Gy in 25–28 fractions to the pelvis) with capecitabine or S-1 and irinotecan is used for nCRT [11]. Several studies from Japan have shown that NAC potentially increases the rate of sphincter preservation and has a similar rate of pathological complete response to nCRT [12–14]. Thus, modified FOLFOX6 plus either bevacizumab or cetuximab is also an option of care as NAC for LARC [12]. Principally, nCRT is used for cases with potentially positive circumferential resection margin (CRM), while NAC is performed for cases with multiple and/or bulky nodal involvement in the mesorectum without CRM involvement. Also, the selective LPLN dissection is simultaneously performed with TME for cases with clinically swollen LPLNs diagnosed by magnetic resonance imaging (MRI) (short axis diameter ≥5 mm) because LPLN can be considered as regional rather than distant (mainly nodes along the internal iliac vessels and obturator nodes) [15]. Prophylactic dissection for cases with non-swollen LPLNs (short axis diameter <5 mm) was not performed. Based on the patients’ tumor findings, physical status, and organ function, the Kyoto University Hospital Colorectal Cancer Care Unit (an experienced multidisciplinary team consisting of surgical oncologists, medical oncologists, gastroenterologists, cancer biologists, and radiologists) discussed and determined the optimal treatment strategy for each patient. The unit also discussed the implementation of adjuvant chemotherapy following the pathological assessment of the resected tumor. According to the Japanese Society for Cancer of the Colon and Rectum (JSCCR) guidelines, rectal cancer patients are usually followed up every 3 months for years 1–3 and every 6 months for years 4–5 by CEA test plus clinic visit, every 6 months for years 1–3 and every 6–12 months for years 4–5 by computed tomography (CT) scan and digital rectal examination, and annual colonoscopy for years 1–3 [16].

### Definitions and statistical analysis

In this study, disease recurrence after surgery was classified into three categories: “local-only,” “distant-only,” and “simultaneous.” Local recurrence was defined as intrapelvic recurrence detected using imaging modalities, including LPLN recurrence and anastomotic recurrence. Distant metastasis was defined as disease recurrence that had spread to remote organs (e.g., the lung, liver, peritoneum, and remote lymph nodes). Only the first disease recurrence in each LARC patient was assessed. Recurrence-free survival (RFS), overall survival (OS), and cumulative incidence of recurrence were plotted using the Kaplan-Meier method. The OS was measured from date of surgery to death. The cumulative incidence of recurrence was assessed in the patients with disease recurrence. Surgically resectable recurrence was defined as a lesion resected with curative-intent, regardless of the use of systemic chemotherapy and/or radiotherapy prior to resection. Best supportive care and/or systemic palliative chemotherapy following disease recurrence was defined as an “unresectable case.” Fisher’s exact test and log-rank tests were used to assess statistical significance (*P*<0.05) for categorical and survival data, respectively. All statistical analyses were performed by RO using JMP Pro ver.15 (SAS Institute, Cary, NC, USA).

## Results

### Patient characteristics

In total, 332 consecutive LARC patients who underwent curative surgery were included in this study. The median follow-up duration was 4.6 years. Clinical stages II and III were observed in 96 (28.9%) and 236 (71.1%) of the patients, respectively (Table 1). Among the 332 patients, 148 patients (44.6%) were upper rectal tumors, 178 (53.6%) were lower rectal tumors, and 6 (1.8%) were anal canal tumors. The median distance from the anal verge was 6cm (interquartile range, 4–8cm; range, 0–15) in this study. Of the 332 patients, 30.1% (*N*=100) received neoadjuvant treatments: nCRT, 15.4% (*N*=51) and NAC, 14.8% (*N*=49). In the surgery alone group (*N*=232), 55.2% of the patients had lesions in the upper rectum, while 92.1% in the nCRT group and 67.3% in the NAC group had lesions in the lower rectum or anal canal. Simultaneous LPLN dissection was performed in 11.2% (*N*=26) of patients in the surgery alone group, 49% (*N*=25) in the nCRT group, and 32.4% (*N*=16) in the NAC group. When compared with the surgery alone group, the nCRT and NAC groups had more clinical T4 cases (18.5% in surgery alone, 33.3% in nCRT, and 28.6% in NAC), as well as clinical stage III (63.8% in surgery alone, 84.3% in nCRT, and 91.8% in NAC). MRI assessment prior to treatment showed that 14.2% (*N*=33) of patients in the surgery alone group, 74.5% (*N*=38) in the nCRT group, and 51.0% (*N*=25) in the NAC group had CRM involvement. In the nCRT group, the abdominoperineal resection (31.4%) or intersphincteric resection (35.3%) was more performed than anterior resection (27.5%). Pathological findings showed that none in the nCRT group had CRM involvement, while 6.1% (*N*=3) in the NAC group and 3.6% (*N*=8) in the surgery alone group.

**Table 1:** Basic characteristics according to treatment prior to surgery for patients with LARC (*N*=332)

	***Overall (N=332)***		***Surgery alone (N=232)***		***nCRT (N=51)***		***NAC (N=49)***	
***Variables***	***N***	***%***	***N***	***%***	***N***	***%***	***N***	***%***
**Median age (range)**	66 (21–90)		68 (28–90)		61 (27–79)		64 (21–81)	
**Gender**								
Male	218	65.7	147	63.4	35	68.6	36	73.5
Female	114	34.3	85	36.6	16	31.4	13	26.5
**Clinical T classification**								
<cT2	35	10.5	32	13.8	2	3.9	1	2.0
cT3	223	67.2	157	67.7	32	62.8	34	69.4
cT4	74	22.3	43	18.5	17	33.3	14	28.6
**Clinical N classification**								
cN0	212	63.9	139	59.9	42	82.4	31	63.3
cN1	92	27.7	72	31.0	7	13.7	13	26.5
cN2	28	8.4	21	9.1	2	3.9	5	10.2
**mr-CRM involvement**	96	28.9	33	14.2	38	74.5	25	51.0
**Clinical stage**								
cStage II	96	28.9	84	36.2	8	15.7	4	8.2
cStage III	236	71.1	148	63.8	43	84.3	45	91.8
**Clinical LPLN swelling**	67	20.2	26	11.2	25	49.0	16	32.4
**Tumor location**^a^								
Upper rectum	148	44.6	128	55.2	4	7.8	16	32.7
Lower rectum	178	53.6	100	43.1	45	88.2	33	67.3
Anal canal	6	1.8	4	1.7	2	3.9	0	0.0
**Surgical procedure**								
Anterior resection	215	64.8	170	73.3	14	27.5	31	63.3
Abdominoperineal resection	52	15.7	26	11.2	16	31.4	0	20.4
Intersphincteric resection	46	13.9	22	9.5	18	35.3	6	12.2
Hartmann procedure	14	4.2	12	5.2	2	3.9	0	0.0
Total pelvic exenteration	5	1.5	2	0.9	1	2.0	2	4.1
**Simultaneous LPLN dissection**	67	20.2	26	11.2	25	49.0	16	32.4
**Pathological T classification**								
p-, ypT0,Tis	17	5.1	1	0.4	12	23.5	4	8.2
p-, ypT1	20	6.0	17	7.3	3	5.9	0	0.0
p-, ypT2	75	22.6	54	23.3	10	19.6	11	22.5
p-, ypT3	184	55.4	129	55.6	24	47.1	31	63.3
p-, ypT4	36	10.8	31	13.4	2	3.9	3	6.1
**Pathological stage**								
0–I	83	25.0	50	21.6	20	39.2	13	26.5
II	129	38.9	89	38.4	22	43.1	18	36.7
III	120	36.1	93	40.1	9	17.6	18	36.7
**Pathological LPLN involvement**	12	3.6	4	1.7	3	5.9	5	10.2
**R0 resection**	321	96.7	224	96.6	51	100	46	93.9
**Pathological CRM involvement**	11	3.3	8	3.6	0	0.0	3	6.1
**Adjuvant chemotherapy**								
None	181	54.5	160	69.0	15	29.4	6	12.2
Dublets^b^	86	25.9	37	16.0	24	47.1	25	51.0
Fluoropyrimidines^c^	62	18.7	34	14.7	11	21.6	17	34.7
Unknown	1	0.9	1	0.4	1	2.0	1	2.0

^a^Defined as follows: upper rectum (between the lower level of the S2 vertebra and the peritoneal reflection), lower rectum (between the peritoneal reflection and the upper level of the puborectal sling), and anal canal (between the upper level of the puborectal sling and the anal verge). (According to the Japanese Classification of Colorectal, Appendiceal, and Anal Carcinoma)[9]

^b^FOLFOX, CAPOX, FOLFIRI, SOX, or IRIS

^c^Infusion 5-FU/LV, capecitabine, or S-1

*Abbreviations*: *LPLN* Lateral pelvic lymph node, *mr-CRM* MRI-assessed circumferential resection margin, *NAC* neoadjuvant chemotherapy, *nCRT* Neoadjuvant chemoradiotherapy

### Sites of recurrence

Overall, 23.5% (*N*=78) of the 332 patients experienced disease recurrence after surgery, and the 3-year recurrence-free survival rate was 76.4%. The most common sites of recurrence were the lung (41.0%, *N*=32), local (38.5%, *N*=30), and liver (23.1%, *N*=18). Although the overall recurrence rate did not differ according to neoadjuvant treatment (23.3% in the surgery alone group, 21.6% in the nCRT group, and 26.5% in the NAC group), the local recurrence rate (18.4%, *N*=9) was higher than the rate of distant metastasis in the NAC group (14.3%, *N*=7) (Table 2). In contrast, all recurrences in the nCRT group (*N* = 11 of 11 patients) were distant metastases (one patient had both distant metastasis and local recurrence). When stratified according to pathological stages, the recurrence rate was 2.0% (*N* = 1 of 50 patients), 20.2% (*N* = 4 of 18), and 37.6% (*N* = 7 of 18) for stages I, II, and III in the surgery alone group, respectively (Table 3). Compared with the surgery alone group, the rate for stage 0-I was higher in the nCRT (10.0%, *N* = 2 of 20; *P*=0.19) and NAC (15.4%, *N* = 2 of 13; *P*=0.11) groups, and these recurrences were all distant metastases (one patient had local recurrence and distant metastasis simultaneously).

**Table 2:** Postoperative disease recurrence in patients with LARC (*N*=332)

	***Overall (N=332)***		***Surgery alone (N=232)***		***nCRT (N=51)***		***NAC (N=49)***	
***Variables***	***N***	***%***	***N***	***%***	***N***	***%***	***N***	***%***
**Overall recurrence**	78	23.5	54	23.3	11	21.6	13	26.5
**Recurrence site**								
Local-only (intrapelvic)	19	5.7	13	5.6	0	0.0	6	12.2
Distant-only	48	14.5	34	14.7	10	19.6	4	8.2
Simultaneous	11	3.3	7	3.0	1	2.0	3	6.1
Organs of first recurrence*								
Local	30	9.0	20	8.6	1	2.0	9	18.4
Lung	32	9.6	21	9.1	6	11.8	5	10.2
Liver	18	5.4	14	6.0	2	3.9	1	2.0
Peritoneum	3	0.9	1	0.4	0	0.0	2	4.1
Others	16	4.8	11	4.7	2	3.9	3	6.1

^*^Multiple organs were allowed

*Abbreviations*: *NAC* Neoadjuvant chemotherapy, *nCRT* Neoadjuvant chemoradiotherapy

**Table 3:** Recurrence pattern according to neoadjuvant treatment according to treatment prior to surgery for patients with LARC (*N*=332)

***Pathological stage***		**Neoadjuvant treatment**		
		***Surgery alone (N=232)***	***nCRT (N=51)***	***NAC (N=49)***
**Stage 0-I**	**Overall**	**2.0% (1/50)**	**10.0% (2/20)**	**15.4% (2/13)**
	Local-only	2.0% (1)	0.0% (0)	0.0% (0)
	Distant-only	0.0% (0)	10.0% (2)	7.7% (1)
	Simultaneous	0.0% (0)	0.0% (0)	7.7% (1)
**Stage II**	**Overall**	**20.2% (18/89)**	**31.8% (7/22)**	**22.2% (4/18)**
	Local-only	3.4% (3)	0.0% (0)	11.1% (2)
	Distant-only	14.6% (13)	27.3% (6)	11.1% (2)
	Simultaneous	2.3% (2)	4.6% (1)	0.0% (0)
**Stage III**	**Overall**	**37.6% (35/93)**	**22.2% (2/9)**	**38.9% (7/18)**
	Local-only	9.7% (9)	0.0% (0)	22.2% (4)
	Distant-only	22.6% (21)	22.2% (2)	5.7% (1)
	Simultaneous	5.4% (5)	0.0% (0)	11.1% (2)

### Resectability of recurrence

**Fig. 1:**
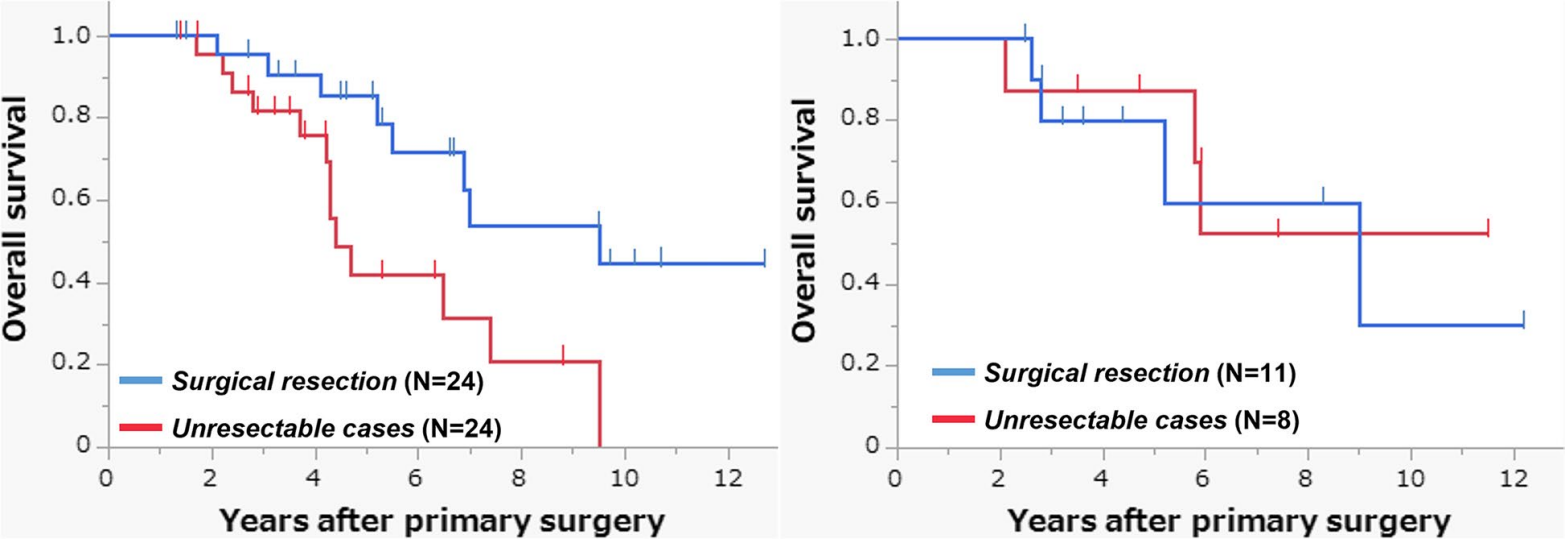
Overall survival after primary surgery according to treatment for recurrence. **A** In patients with distant-only recurrence (*N*=48). **B** In patients with local-only recurrence (*N*=19)

Curative-intent resection of recurrent lesions was successfully performed in 46% (*N*=36) of patients with recurrence. Of the 78 patients with recurrence, 61.5% (*N*=48) had distant-only recurrence (mostly lung [*N*=26] or liver [*N*=12] only), and curative-intent resection was performed in 50.0% (*N*=24) of them. Also, 24.4% (*N*=19) of the patients with recurrence had local-only recurrences and 57.9% (*N*=11) underwent curative-intent salvage surgery. However, among the 11 patients who had both distant metastasis and local recurrence simultaneously, only 1 patient (9.1%) underwent curative-intent surgery. Among the patients with distant-only recurrence, the resection rate was similar according to neoadjuvant treatment (50.0% in the surgery alone group [*N* = 17 of 34 patients], 50.0% in the nCRT group [*N* = 5 of 10], and 50.0% in the NAC group [*N* = 2 of 4]). Also, among the patients with local-only recurrence, the rate of salvage surgery in the surgery alone group (61.5%, *N* = 8 of 13 patients) did not largely differ from that in the NAC group (50.0%, *N* = 3 of 6) (no local-only recurrence in the nCRT group). In terms of survival in patients with recurrence, curative-intent resection for patients with distant metastases showed a significantly longer OS after primary surgery than the unresectable cases (HR [95%CI], 0.34 [0.14–0.84]; *P*=0.019; Fig. 1A). However, for the patients who had local-only recurrence, salvage surgery did not affect patients’ OS after primary surgery (HR [95%CI], 1.23 [0.27–5.69]; *P*=0.784; Fig. 1B). These findings were also observed for OS after recurrence (Supplementary Fig. 1A and B). When divided according to neoadjuvant treatment, surgical resection of distant metastasis was beneficial, even in patients who received nCRT or NAC preoperatively (Supplementary Fig. 1C).

### Cumulative incidence of recurrence

**Fig. 2:**
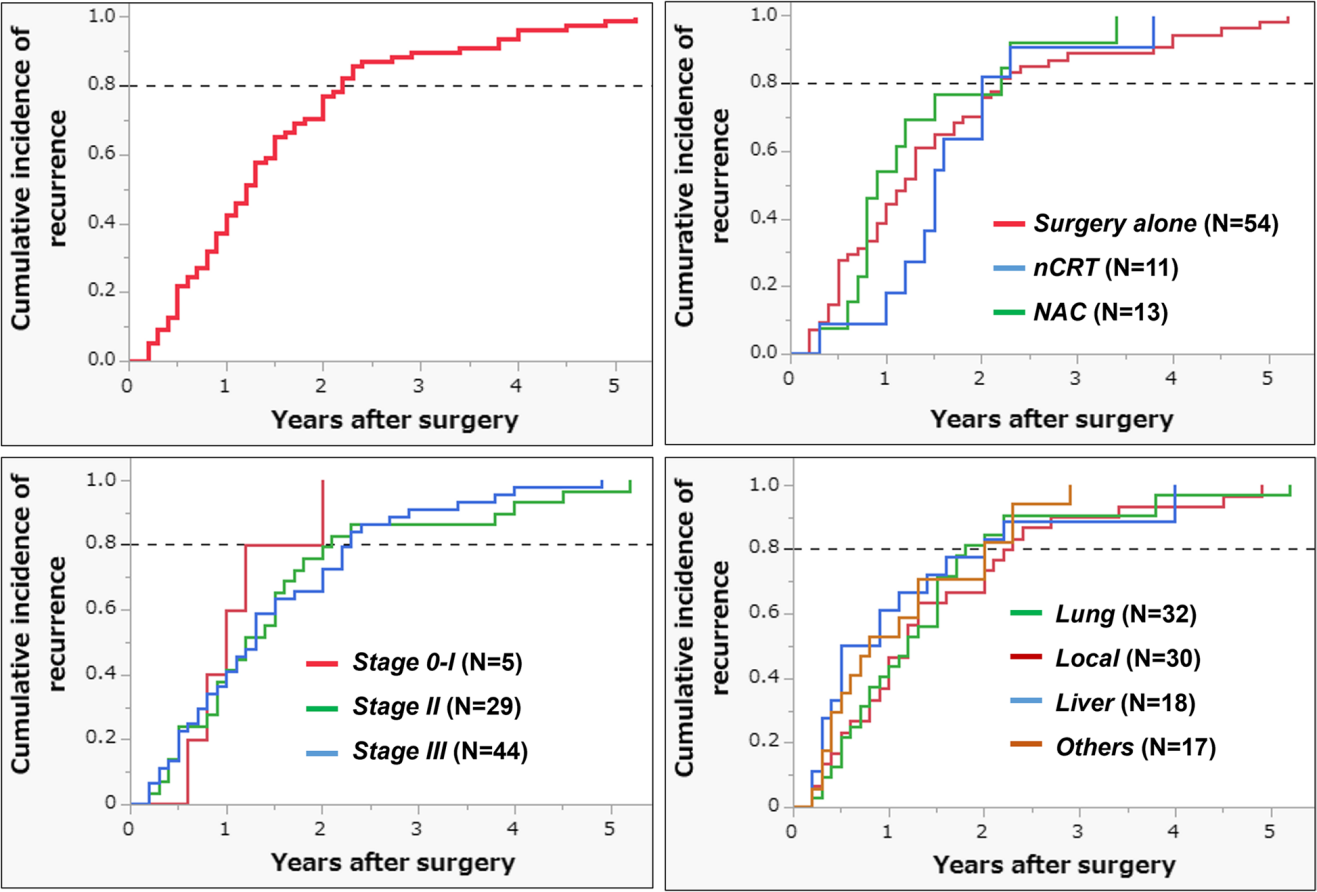
Cumulative incidence of disease recurrence after surgery for LARC. **A** Overall patients with recurrence (*N*=78). **B** According to neoadjuvant treatments (surgery alone [*N*=54], nCRT [*N*=11], and NAC [*N*=13]). **C** According to pathological stages (0-I [*N*=5], II [*N*=29], and III [*N*=44]). **D** According to recurrence sites (lung [*N*=32], local [*N*=30], liver [*N*=18], and others [*N*=17]). Multiple sites were allowed

Following radical surgery for LARC, adjuvant chemotherapy was administered in 30.7% (*N*=71) of patients in the surgery alone group, 85.7% (*N*=42) in the NAC group, and 68.7% (*N*=35) in the nCRT group (Table 1). The cumulative incidence of recurrence is shown in Fig. 2A. The curve showed that 80% of the recurrences occurred 2.2 years after surgery. In this study, 98.7% of the recurrences occurred 5 years after surgery. Even when stratified according to neoadjuvant treatment, over 80% of the recurrences occurred within 2.2 years: NAC, 84.6%; nCRT, 81.8%; and none, 81.5% (Fig. 2B). Similarly, over 80% of the recurrences occurred within 2.2 years regardless of the pathological stage (Fig. 2C) or site of recurrence (Fig. 2D). According to the recurrence pattern, 83.3% of the patients who had distant-only recurrence occurred within 2.2 years and 84.2% of those who had local-only recurrence occurred within 2.7 years (Supplementary Fig. 2). All simultaneous recurrences (both distant metastasis and local recurrence detected simultaneously) occurred 2 years postoperatively.

## Discussion

This study evaluated the postoperative recurrence patterns of LARC in a real-world setting and found that the cumulative incidence and resectability of recurrence were similar regardless of neoadjuvant treatment. However, patients with LARC who receive neoadjuvant treatment and whose tumors are downstaged to pathological stage 0-I should be carefully followed-up for distant metastasis. Furthermore, the curative-intent resection of distant metastasis affected the OS of patients with LARC, emphasizing the importance of intensive imaging surveillance for detecting distant metastasis in the early postoperative phase.

The postoperative recurrence rate and 3y-RFS in patients with LARC were 23.5% and 76.4%, respectively; local recurrence and distant metastasis occurred in 9% and 17.8%, respectively. The recurrence rate was similar regardless of neoadjuvant treatment (23.3% in the surgery alone group, 26.5% in the NAC group, and 21.6% in the nCRT group); however, the local recurrence rate in the NAC group (18.4%) was higher than that in the other groups (8.6% in the surgery alone group and 2.0% in the nCRT group), and 69.2% of the recurrences in the NAC group had local recurrence. We introduced NAC for patients with LARC based on our multicenter Phase-2 study that demonstrated comparable rates of R0 resection (98%) and pathologic complete response (17%) of neoadjuvant mFOLFOX6 plus bevacizumab or cetuximab to historical data of nCRT [12]. Although we used this strategy as an option until 2015, further investigation for locoregional control in this trial is needed. In contrast, all recurrences in the nCRT group were accompanied with distant metastases. The PRODIGE 23 trial showed a reduction in the risk of distant metastasis without locoregional failure using the TNT strategy (FOLFIRINOX followed by nCRT), suggesting that administering preoperative systemic chemotherapy might control distant metastasis during the time interval between nCRT and surgery in patients with LARC [17]. However, neoadjuvant treatments are associated with an increased risk of surgical complications and fecal incontinence [18, 19]. Yamamoto et al. reported that TME alone was potentially sufficient for LARC without MRI-assessed CRM (mr-CRM) involvement or swollen LPLN because of the low recurrence rate in the pelvic cavity (2.2%) and LPLN (1.9%) [20]. An optimal personalized strategy using these preoperative treatments should be established for each LARC case.

We found a high risk of distant metastasis in stage 0-I patients who received neoadjuvant treatment (15.4% in the NAC group and 10.0% in the nCRT group) (Table 3). Previous studies reported that the disease recurrence rate in pathological stage I rectal cancer without preoperative treatment was 3.7–10.0% [21–23]. A Japanese nationwide cohort showed a 6.9% recurrence rate in surgical case of stage I rectal cancer [24]. Thus, we believe that postoperative imaging is important even in patients with stage I rectal cancer with preoperative treatment whose tumors were successfully downstaged from LARC, although the current ASCO and NCCN guidelines do not recommend routine surveillance for early-stage cancer [5, 25].

In this study, a curative-intent resection rate of 50% or higher was observed in both distant-only recurrence (50.0%) and local-only recurrence (57.9%) patients. Not unexpectedly, the rate was only 9.1% in patients with both distant metastasis and local recurrence simultaneously. Regarding the impact of surgical resection of recurrence on patients’ survival, surgical resection of distant metastasis significantly improved OS (HR for OS from primary surgery [95%CI], 0.34 [0.14–0.84]; Fig. 1A). Previous studies have also suggested that the early detection of hematogenous recurrences, such as lung and liver metastases, can often lead to curative-intent resections and improve patient survival [26–29]. Thus, routine postoperative surveillance using imaging modalities is important for detecting hematogenous recurrence, especially in the early postoperative phase. In contrast, salvage surgery for local recurrence did not improve OS (HR for OS from primary surgery [95%CI], 1.23 [0.27–5.69]; Fig. 1B). This finding is consistent with that of a previous report by Ikoma et al. Salvage surgery for local recurrence should be carefully considered because it often requires extended radical resection with a high risk of morbidity or declining quality of life [30].

We further report that >80% of recurrences occur within 2.2 years and all recurrences (except for one case with lung metastasis) occurred within 5 years after surgery, regardless of neoadjuvant treatment, pathological stage, or recurrence site (Fig. 2), in addition to recurrence pattern (Supplementary Fig. 2). These findings indicate that neoadjuvant treatment does not affect the doubling time of recurrent lesions; because the TNT strategy has recently been used increasingly for LARC, further assessment is required. Notably, the JSCCR guidelines recommend a shorter interval at years 1–3 after surgery, which is the same as that for colon cancer or early rectal cancer, while the ASCO, ESMO, and NCCN guidelines handle advanced rectal cancers separately from colon cancer or early-stage cancer and recommend a shorter interval at years 1–2 after surgery [31–34]. Although the optimal intensity and frequency of surveillance remain undefined owing to the lack of robust evidence, we believe that the distribution of recurrences is important for cost-effective surveillance strategies [35].

This study had several limitations. First, although the surgical strategy and postoperative surveillance were largely consistent throughout the study period in this single-institutional study, there might be some differences between the earlier and later cases. Also, the clinical decision regarding neoadjuvant therapy and surgery for recurrence was associated with patient or surgeon selection. This study did not compare oncological outcomes according to neoadjuvant treatments after adjusting background confounding factors. Further studies with larger numbers of patients are required.

In conclusion, regardless of neoadjuvant treatment, >80% of recurrences occur 2.2 years after surgery. Even if neoadjuvant treatment can downstage LARC to pathological stage 0-I, careful follow-up is needed. Detecting asymptomatic distant metastases using intensive surveillance must be beneficial for improving the survival of patients with LARC. In contrast, detecting asymptomatic local recurrence remains controversial because salvage surgery for local recurrence does not affect patients’ survival. Therefore, it is important to reduce local recurrence with sufficient surgical strategy.

### Supplementary Information

**Additional file 1: Fig. S1.** Overall survival after recurrence detected in LARC patients with disease recurrence. A. According to treatment for recurrence in patients with distant-only recurrence (*N*=48). B. According to treatment for recurrence in patients with local-only recurrence (*N*=17). C. According to neoadjuvant treatments in patients who had distant-only recurrence and underwent surgical resection of distant metastasis (*N*=24). **Fig. S2.** Cumulative incidence of disease recurrence following TME surgery according to recurrence patterns (local-only recurrence [*N*=19], distant-only recurrence [*N*=48], and simultaneous recurrences [*N*=11]).

## Data Availability

The datasets used and/or analyzed during the current study are available from the corresponding author on reasonable request.

## Abbreviations

NAC: Neoadjuvant chemotherapy
nCRT: Neoadjuvant chemoradiotherapy
