# Supplementary material for: Postoperative recurrence in locally advanced rectal cancer: how does neoadjuvant treatment affect recurrence pattern?
Source: World J Surg Oncol. 2023 Aug 16;21:247. doi: 10.1186/s12957-023-03136-0 (PMC10428603; doi:10.1186/s12957-023-03136-0)

**Supplementary Files**

**Supplementary Figure 1**. Overall survival after recurrence detected in LARC patients with disease recurrence.

**A**. According to treatment for recurrence in patients with distant-only recurrence (N=48). **B**. According to treatment for recurrence in patients with local-only recurrence (N=17). **C**. According to neoadjuvant treatments in patients who had distant-only recurrence and underwent surgical resection of distant metastasis (N=24).

**
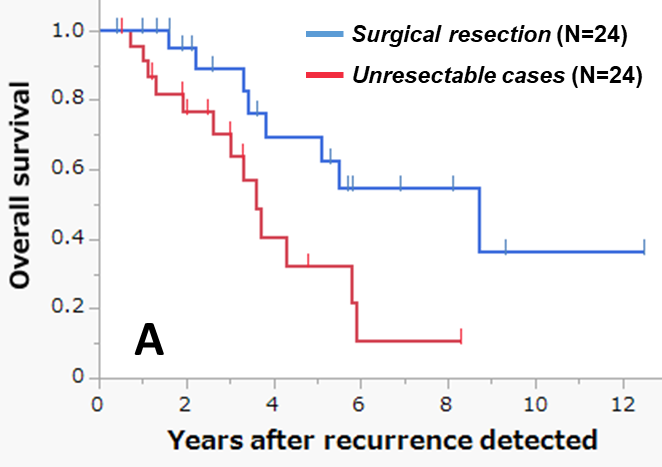

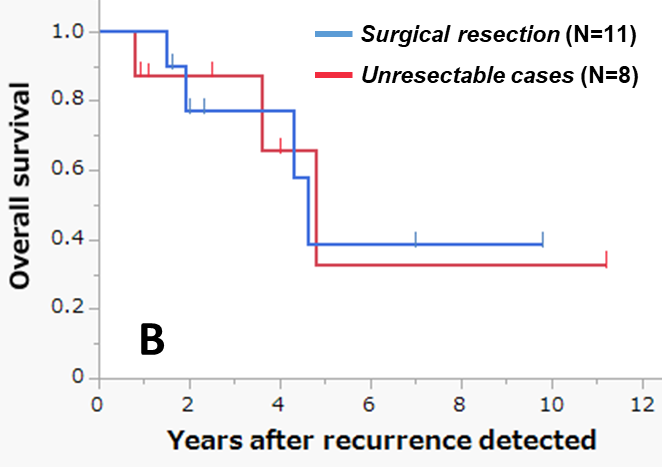
**

**
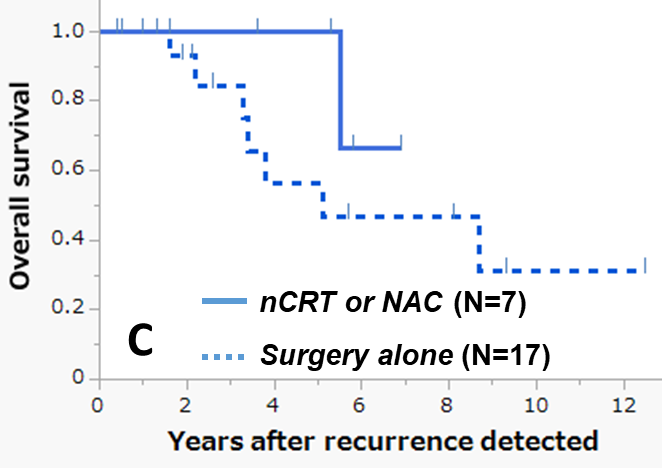
**

**Supplementary Figure 2**. Cumulative incidence of disease recurrence following TME surgery according to recurrence patterns (local-only recurrence [N=19], distant-only recurrence [N=48], and simultaneous recurrences [N=11]).


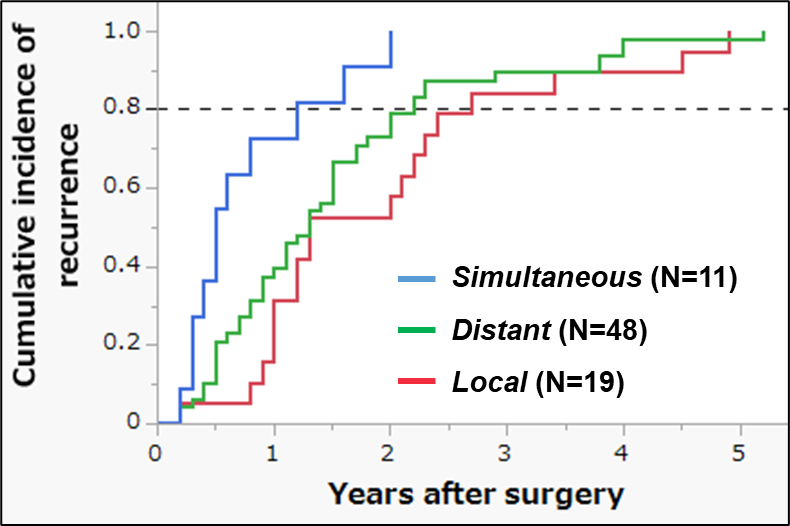

Supplement: Supplementary file 1 — Additional file 1: Fig. S1. Overall survival after recurrence detected in LARC patients with disease recurrence. A. According to treatment for recurrence in patients with distant-only recurrence (N=48). B. According to treatment for recurrence in patients with local-only recurrence (N=17). C. According to neoadjuvant treatments in patients who had distant-only recurrence and underwent surgical resection of distant metastasis (N=24). Fig. S2. Cumulative incidence of disease recurrence following TME surgery according to recurrence patterns (local-only recurrence [N=19], distant-only recurrence [N=48], and simultaneous recurrences [N=11]). [file 12957_2023_3136_MOESM1_ESM.docx]
